# Protected Organic Acid and Essential Oils for Broilers Raised Under Field Conditions: Intestinal Health Biomarkers and Cecal Microbiota

**DOI:** 10.3389/fphys.2021.722339

**Published:** 2021-10-25

**Authors:** Cristiano Bortoluzzi, Ludovic Lahaye, Jarred Oxford, Derek Detzler, Cinthia Eyng, Nicolle Lima Barbieri, Elizabeth Santin, Michael Henry Kogut

**Affiliations:** ^1^Jefo Nutrition Inc., Saint-Hyacinthe, QC, Canada; ^2^Department of Animal Science, Western Paraná State University, Marechal Cândido Rondon, Brazil; ^3^Poultry Diagnostic and Research Center, University of Georgia, Athens, GA, United States; ^4^United States Department of Agriculture – Agricultural Research Service (USDA-ARS), Southern Plains Agricultural Research Center, College Station, TX, United States

**Keywords:** antimicrobial resistance genes, biomarkers, broiler, essential oils, intestinal inflammation, organic acids

## Abstract

The objective of the present study was to evaluate the effect of protected organic acids (OA) and essential oils (EO) [P(OA + EO)] on the intestinal health of broiler chickens raised under field conditions. The study was conducted on four commercial farms. Each farm consisted of four barns, two barns under a control diet and two tested barns supplemented with P(OA + EO), totaling 16 barns [8 control and 8 under P(OA + EO)]. The control group was supplemented with antibiotic growth promoters [AGP; Bacitracin Methylene Disalicylate (50 g/ton) during starter, grower and finisher 1, and flavomycin (2 g/ton) during finisher 2]. The tested group was supplemented with 636, 636, 454, and 454 g/ton of P(OA + EO) during starter, grower, finisher 1 and 2, respectively. Eighty birds were necropsied (40/treatment; 20/farm; and 5/barn) to collect blood, jejunal tissue, and cecal contents. The data were submitted to analysis of variance (ANOVA) (*P* < 0.05) or Kruskal-Wallis’ test and the frequency of antimicrobial resistant (AMR) genes was analyzed by Chi-Square test (*P* < 0.05). It was observed that the supplementation of P(OA + EO) reduced (*P* < 0.05) the histopathology scores, such as the infiltration of inflammatory cells in the epithelium and lamina propria and tended (*P* = 0.09) to reduce the serum concentration of calprotectin (CALP). The supplementation of P(OA + EO) reduced the serum concentration of IL-12 (*P* = 0.0001), IL-16 (*P* = 0.001), and Pentraxin-3 (*P* = 0.04). Additionally, P(OA + EO) maintained a cecal microbiota similar to birds receiving AGP. The substitution of AGP by P(OA + EO) reduced (*P* < 0.05) the frequency of four AMR genes, related to gentamicin (three genes), and aminoglycoside (one gene). Overall, the inclusion of P(OA + EO), and removal of AGP, in the diets of commercially raised broiler chickens beneficially changed the phenotype of the jejunum as shown by the lowered ISI scores which characterizes an improved intestinal health. Furthermore, P(OA + EO) significantly reduced the serum concentration of several inflammatory biomarkers, while maintaining the diversity and composition of the cecal microbiota similar to AGP fed chickens and reducing the prevalence of AMR genes.

## Introduction

Optimal intestinal health in animal production is of paramount importance for an animal to achieve its genetic potential and is strongly correlated with overall health and welfare. However, several physiological functions must be studied to define a ‘‘healthy intestine’’ including nutrient digestion and absorption, metabolism and energy generation, a stable microbiome, mucus layer development, barrier function, and mucosal immune responses (Swaggerty et al., 2021)^1^. Organic acids (OA) and essential oils (EO) represent alternatives to antibiotic growth promoters (AGP) used in animal production because they can improve growth performance by different mechanisms. These compounds may have bacteriostatic and bactericidal properties (Ricke, 2003), or direct effects on the host by improving the development of the gastrointestinal tract (GIT) and modulating the immune system. The immunomodulatory effects of OA and EO includes induction of Toll-like receptors, and induction of proliferation and maturation of T-Helper cells (Th-1 and Th-2) to maintain a balance between cellular and humoral immune response (Hashemi and Davoodi, 2012) which may lead to improved growth performance and other health related paraments (Chowdhury et al., 2009; Islam, 2012).

Organic acids and EO are volatile and can evaporate quickly during feed processing or being absorbed in the stomach and proximal small intestine (Michiels et al., 2008). Microencapsulation is a feasible strategy to be used to, among other advantages, improve stability and protect feed additives during processing, reduce the effective dose, delay the absorption of these molecules and to be slowly released throughout the intestine of the animals (Choi et al., 2020). For instance, Choi et al. (2020) showed that 15.5% of microencapsulated thymol was released in the stomach, 41.85% was released in the mid-jejunum section, and 2.21% was recovered in the feces, showing that lipid matrix microparticles can maintain the stability of thymol and allowed a progressive release of thymol in the intestine of pigs. Therefore, microencapsulated OA and EO may have more influence on the distal portions of the GIT when compared to non-encapsulated molecules which may be essential to prevent pathogen proliferation in the lower parts of the intestine, where higher bacterial concentration is found.

The supplementation of OA and EA provide synergistic effects to improve growth performance and gut health of animals (Stefanello et al., 2019; Yang et al., 2019). Yang et al. (2019) demonstrated that a mixture of sorbic acid, fumaric acid, and thymol modulated the microbiota, increased sucrase and maltase activities in the jejunal mucosa, and increased the expression of tight junction protein genes which reflected in a better feed efficiency in broiler chickens. Stefanello et al. (2019) showed that a combination of microencapsulated fumaric, citric, malic, and sorbic acids plus thymol, vanillin, and eugenol increased the expression of tight junction protein genes, improved nutrient digestibility, and intestinal health of broilers, showing that this blend is beneficial for AGP free programs. Furthermore, biomarkers of intestinal health that should be reliable, minimally invasive, and easy to process are constantly being searched (Dal Pont et al., 2021). Among these, calprotectin (CALP), a protein released by heterophils and macrophages during inflammation, has been shown to be a promising biomarker detected in serum or excreta of chickens (Dal Pont et al., 2021). Associated with other approaches, CALP measurement may add essential information when discussing intestinal health of poultry flocks, especially when field samples are analyzed. Therefore, the hypothesis of this study was that the supplementation of protected OA and EO—P(OA + EO)—would improve the intestinal health, modulate the cecal microbiota, and reduce the prevalence of antimicrobial resistant (AMR) genes in the microbiota of broiler chickens. The objective of the present study was to evaluate the dietary supplementation of P(OA + EO) on the jejunum histopathology, serum cytokines and CALP concentrations, microbiota diversity and composition, and frequency of AMR genes in the cecal microbiota of broiler chickens raised under field conditions.

## Materials and Methods

### Birds, Housing, and Treatments

The study was conducted on four commercial farms. Each farm consisted of four barns, two barns under a control diet and two tested barns supplemented with P(OA + EO), totaling 16 barns [8 control and 8 under P(OA + EO)] supplementation. The control group was supplemented with antibiotic growth promoter [AGP; bacitracin methylene disalicylate (BMD; 50 g/ton) during starter, grower and finisher 1, and flavomycin (2 g/ton) during finisher 2]. The tested group was supplemented with 636, 636, 454, and 454 g/ton of P(OA + EO) during starter, grower, finisher 1 and 2, respectively. Both groups were supplemented with narasin (63 and 72 g/ton, for grower and finisher 1, respectively), vaccinated against coccidiosis (ADVENT^®^) at the hatchery, and followed by a withdrawal period of AGP and narasin at the end of the production cycle. The P(OA + EO) consists of fumaric, citric, malic, and sorbic acids plus thymol, vanillin, and eugenol microencapsulated in a matrix of triglycerides from hydrogenated vegetable oil (Jefo Nutrition Inc., Saint-Hyacinthe, QC, Canada).

### Samples Collected

A total of 80 broiler chickens were euthanized by cervical dislocation, and necropsied (40 birds/treatment group, being 20 birds/farm and 5 birds/barn) to collect a section of the jejunum for *I See Inside* (ISI) analysis, blood to determine the serum concentration of cytokines array and CALP, and cecal content to analyze the diversity and composition of the microbiota and the frequency of AMR genes. The sampling was performed on farms 1 (20 birds) and 2 (20 birds) at 28- and 25-days old broiler chickens, and on farms 3 (20 birds) and 4 (20 birds) at 27- and 25-days old broiler chickens, respectively. Since the birds were not sampled on the exact same age because of the date differences in the beginning of the trial, each farm was considered as a block during the statistical analyses.

### *I See Inside*—Histopathological Analysis

A section of jejunum (∼2 cm) was collected from each bird, rinsed with phosphate buffer solution (PBS), and immersed into formalin 10% for fixation. The samples were then embedded in paraffin following common histological routine and stained with hematoxylin and eosin.

For microscopic evaluation, the *I See Inside* (ISI) methodology was used to determine histologic alterations in the jejunum (Belote et al., 2019), and 20 intact villi/birds were evaluated. Briefly, the ISI methodology is based on a numerical score of alteration. An impact factor (IF) is defined for each alteration in the microscopic analysis, according to the reduction of the organ functionality. The IF ranges from 1 to 3, with three being the worst impacting organ function. The parameters evaluated by the ISI method in the intestine are lamina propria thickness, epithelial thickness, proliferation of enterocytes, inflammatory cell infiltration on the epithelium, inflammatory cell infiltration in the lamina propria, increase of goblet cell, congestion and presence of *Eimeria* oocysts.

In addition, the score of 0–3 is based on the intensity of the observed lesion: score 0 (absence of lesion), score 1 (alteration of up to 25% of the area), score 2 (alteration of 25–50% of the area), and score 3 (alteration of more than 50% of the area). To obtain the final value of the ISI index, the IF of each alteration is multiplied by the respective score number, according to the formula ISI = Σ(IF × S), where IF = impact factor and S = Score.

### Serum Calprotectin Concentration

The serum concentration of CALP was determined by an ELISA commercial kit (MBS1601938) following the manufacturer recommendations (MyBiosource Inc., San Diego, CA, United States). Briefly, 40 μL of serum was incubated with anti-CALP antibody and streptavidin-HRP for 60 min at 37°C. The samples were then washed with buffer for five times and incubated in dark room with 50 μL of “solution B” for 10 min at 37°C. Lastly, 50 μL of Stop Solution was added, and the optical density was determined using a microplate reader set to 450 nM. The concentration of each sample was determined based on the standard curve.

### Chicken-Specific Cytokine Array Analysis

The cytokines serum concentration was measured by a chicken specific cytokine array (Quantibody^®^ Chicken Cytokine Array 1) following the manufacturer recommendations (RayBiotech, Norcross, GA, United States). The concentration of IFN-gamma, IL-6, IL-10, IL-12, IL-16, pentraxin 3 (PTX3), and chemokine ligand 5 (CCL5) was determined. This sandwich ELISA-based quantitative array platform allows the determination of the concentration of multiple cytokines simultaneously. Briefly, a capture antibody is bound to a glass array surface. After incubation with the sample, the target cytokine is bound on the solid surface. A second biotin-labeled detection antibody is then added, which can recognize a different epitope on the target cytokine. The cytokine-antibody-biotin complex can be visualized by the addition of streptavidin-conjugated Cy3 equivalent dye, using a laser scanner (InnoScan 710 Microarray Scanner; Innopsys Inc., Chicago, IL, United States).

### Cecal Microbiota Analysis—16S rRNA Sequencing and Bioinformatics

The caeca from each sampled bird were collected and placed into a Ziploc bag, immediately put in ice, and taken to the lab. The cecal content was gently squeezed into a 10 mL cryotube and frozen at −80°C for further analysis of the cecal microbiota.

The sample preparation was done in accordance with (Bortoluzzi et al., 2018). An amount of 200 μg of the content was used for DNA isolation following the manufacturer recommendations (PowerViral Environmental RNA/DNA Isolation Kit—Mo Bio; Qiagen, Carlsbad, CA, United States). DNA was then quantified using the Qubit^TM^ 4 Fluorometer (Thermo Fisher Scientific). V3–V4 region of the 16S rRNA gene was amplified using the primer set S-D-Bact-0341-b-S-17/S-D-Bact-0785-a-A-21 (Klindworth et al., 2013). Polymerase chain reaction (PCR) products were purified with a magnetic bead-based clean-up system (Agencourt AMPure XP; Beckman Coulter, Brea, CA, United States). Indexed libraries were prepared by limited-cycle PCR using Nextera technology and further cleaned up with AMPure XP magnetic beads (Beckman Coulter). Libraries were pooled at equimolar concentrations (4 nM), denatured, diluted, and loaded onto the MiSeq flow cell. Sequencing on Illumina MiSeq platform was performed by using a 2 × 250 bp paired end protocol, according to the manufacturer’s instructions (Illumina, San Diego, CA, United States).

Paired-end sequenced reads of samples were analyzed combining PANDAseq2 and the wrapper package Quantitative Insights Into Microbial Ecology (QIIME) v1 (Caporaso et al., 2010; Masella et al., 2012). High-quality reads were binned into operational taxonomic units (OTUs) at a 97% similarity threshold using UCLUST (Edgar, 2010). For bacterial taxonomy assignment, Greengenes database from May 2013 release^2^ was used. The chimera filtering was performed discarding singleton OTUs. Subsampling to the number of sequences in the sample with the least coverage was not performed to correct for different sequencing depth of each sample; however, samples that had less than 10,000 reads after Illumina MiSeq sequencing were re-sequenced. The bacterial abundance data were imported into R (version 3.6.2) on Rstudio v1.1.463 where all statistical analysis were performed using R package *phyloseq* (McMurdie and Holmes, 2013; Callahan et al., 2016). Taxa that were present in less than one sample were excluded from the present analysis using the *decontam* R package (Davis et al., 2018).

### Frequency of Antimicrobial Resistance Genes

All PCR analysis for characterizing AMR genes was carried out using the following protocol with minor modifications for annealing temperatures of the primers. Briefly, DNA samples were amplified using PCR in multiplex panels to amplify a series of the common AMR genes (Johnson et al., 2008) harbored by Enterobacteriaceae species.

All PCR reactions were prepared in a total volume of 25 μL for each sample. Components for a PCR reaction consisted of 2.5 μL of 10× PCR buffer, 0.4 μL of 10 mM MgCL_2_, 1.25 μL of (0.2 M) dNTP mixture, 2 μL of TAQ (Dream TAQ, Thermo Fisher Scientific), 1.4 μL of primer pool, 2 μL of DNA, and 15.45 μL of sterile molecular grade water. Positive control strains were included in the analysis for the appropriate genes of interest from previously characterized strains in our lab collection and negative controls included sterile water in place of DNA. Amplification parameters of the thermocycler (Mastercycler X50, Eppendorf, Hamburg, Germany) included an initial denaturing step at 95°C for 10 min, followed by 30 rounds of [94°C for 30 s (various annealing temperatures), for 30 s, 68°C for 3 min], with a final extension of 72°C for 10 min and a final hold of 4°C.

Polymerase chain reaction products generated were subjected to electrophoresis which was performed in a 2% agarose gel (Agarose LE, Lonza, Alpharetta, GA, United States) running at 100 V for 90 min. The gel was stained with ethidium bromide (0.25%) solution for 20 min, visualized using an imager (UVP BioDock-It^2^ Imager, Analytik Jena, Jena, Germany) and analyzed for the presence of PCR products of the appropriate size when compared with control strains for the targeted gene.

### Statistical Analysis

The ISI data were analyzed by the non-parametric Kruskal-Wallis’s test using SAS 9.4 (*P* < 0.05). The serum concentration of CALP and cytokines was checked for normality (Shapiro-Wilk’s test) and homogeneity of variances (Bartlett’s test), and then submitted to analysis of variance (ANOVA) (*P* < 0.05) using SAS 9.4. Each farm was considered as a block during the analysis. The differences in alpha diversity were evaluated, based on the data distribution of metrics, using ANOVA normally distributed data or Wilcoxon-Mann-Whitney with Holm-Bonferroni correction method for non-normally distributed data. To compare microbial composition between samples, beta-diversity was measured by calculating the weighted or unweighted UniFrac distance matrix. Principal coordinates analysis (PCoA) was applied on the distance matrices to generate bi-dimensional plots on R. The frequency of the main bacterial families observed was submitted to a non-parametric one-way ANOVA (Kruskal-Wallis test). A *P* < 0.05 after false discovery rate (FDR) correction was considered as statistically significant. The prevalence of AMR genes was submitted to a Chi-Square test (*P* < 0.05).

## Results

### *I See Inside*—Histopathological Analysis

**FIGURE 1:**
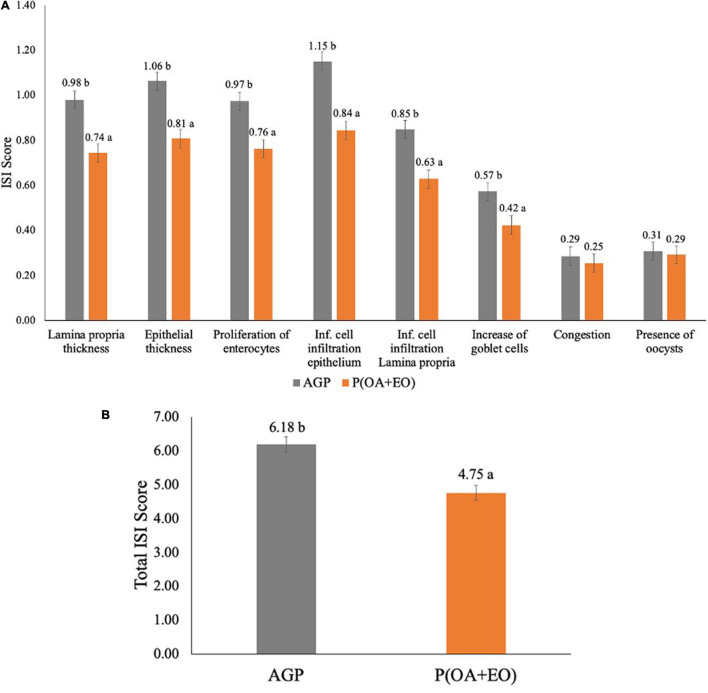
Histopathological analysis by score **(A)** and total **(B)** of the jejunum of broiler chickens supplemented with antibiotic growth promoter (AGP) or protected organic acids (OA) and essential oils (EO) [P(OA + EO)] raised under field conditions. Values are means of 40 replicates. Inf, inflammatory [± standard error of mean (SEM); ^*ab*^*P* < 0.05].

The results of the ISI analysis are shown in Figure 1. It was observed that the supplementation of P(OA + EO) significantly reduced most of the parameters evaluated (Figure 1A) such as lamina propria thickness (*P* = 0.002), epithelial thickness (*P* = 0.006), proliferation of immature enterocytes (*P* = 0.040), inflammatory cell infiltration in the epithelium (*P* = 0.001) and in the lamina propria (*P* = 0.001) and the increase of goblet cells (*P* = 0.005). Lastly, the supplementation of P(OA + EO) reduced the total ISI score (*P* = 0.001; Figure 1B), compared to the AGP fed birds.

### Serum Calprotectin and Chicken-Specific Cytokine Array Analyses

The results of the serum concentration of CALP and the cytokine array are shown in Table 1. It was observed that the supplementation of P(OA + EO) tended to reduce (*P* = 0.090) the serum concentration of CALP compared to AGP fed chickens. Moreover, the supplementation of P(OA + EO) reduced the serum concentration of IL-12 (*P* = 0.001), IL-16 (*P* = 0.001), and PTX3 (*P* = 0.010), and tended to reduce IL-10 (*P* = 0.070). Even though not statistically different, it is worth it to note that the supplementation of P(OA + OA) numerically decreased the serum concentration of IFN-gamma.

**TABLE 1:** Serum concentration of calprotectin (ng/ml) and cytokines (pg/ml) of broiler chickens supplemented with antibiotic growth promoter (AGP) or protected organic acids (OA) and essential oils (EO) [P(OA + EO)] raised under field conditions.

**Treatment**	**CALP**	**IFN-g**	**IL-6**	**IL-10**	**IL-12**	**IL-16**	**PTX3**	**CCL5**
AGP	30.1	151.6	805.0	160.9	15.3^b^	57.6^b^	87.3^b^	127.4
P(OA + EO)	23.8	118.9	867.6	112.5	5.0^a^	22.7^a^	60.5^a^	122.7
SEM	2.68	19.8	138.0	20.4	2.08	6.9	8.56	13.2
*P*-value	0.09	0.30	0.81	0.07	0.0007	0.0002	0.01	0.67

*Values are means of 40 replicates.*

*CALP, calprotectin; PTX3, pentraxin 3; CCL5, chemokine ligand 5; SEM, standard error of mean. ^*ab*^*P* < 0.05.*

### Cecal Microbiota: Alpha and Beta Diversity

**FIGURE 2:**
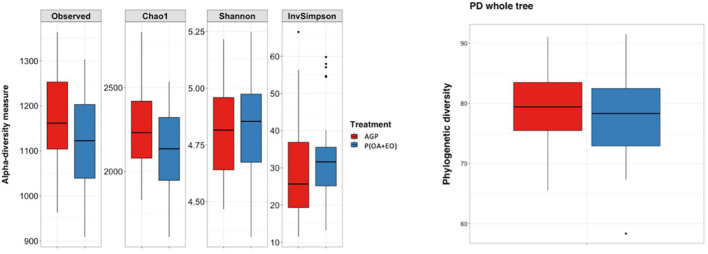
Alpha diversity indices of the cecal microbiota of broiler chickens supplemented with antibiotic growth promoter (AGP) or protected organic acids (OA) and essential oils (EO) [P(OA + EO)] raised under field conditions. Values are means of 40 replicates. PD, phylogenetic diversity (Observed species: *P* = 0.048; Chao1: *P* = 0.048; Shannon, InvSimpson, and PD whole tree: *P* > 0.05).

The results of the alpha diversity indices are shown in Figure 2. The analysis reported significant differences between groups when comparing the number of Observed Species (*P* = 0.048) and Chao1 (*P* = 0.048) indices. It shows that the bacteria present are similar in the phylogenetic relationships of taxa within each microbiota, in the evenness of taxa distribution (no specific dominance of a bacterial group) across treatments but are slightly different in the richness (total number of a specific bacterial group) of taxa.

**FIGURE 3:**
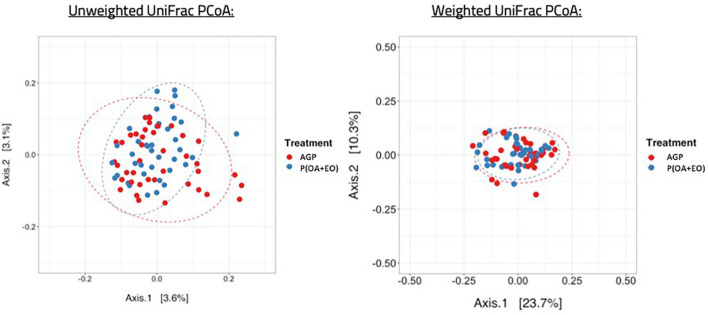
Beta diversity of the cecal microbiota of broiler chickens supplemented with antibiotic growth promoter (AGP) or protected organic acids (OA) and essential oils (EO) [P(OA + EO)] raised under field conditions. Values are means of 40 replicates (Unweighted UniFrac: *P* = 0.019; Weighted UniFrac: *P* > 0.05).

Regarding the beta diversity (Figure 3), the microbiota of AGP and P(OA + EO) fed birds shared a common dispersion of data, therefore, their variances are homogeneous. To summarize the differences between ecological communities, we applied the Unique Fraction method (UniFrac) to define if qualitative differences (unweighted UniFrac) or quantitative differences (weighted UniFrac) were present among groups based on phylogenetic relationships (UniFrac considers phylogenetic relationships among taxa as central information). The results from this analysis show that both treatments led to a separation of groups in distinct clusters only when applying the unweighted UniFrac method. The permutational multivariate analysis of variance (PERMANOVA) applied to the distance matrix of unweighted UniFrac showed a significant difference of the centroids of the clusters of samples (*P* = 0.019). These results indicate that the differences rely on bacteria presence or absence and not on their relative abundance.

### Cecal Microbiota: Composition

**FIGURE 4:**
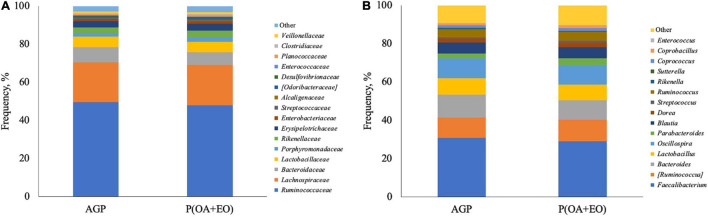
Composition of the cecal microbiota at family **(A)** and genus **(B)** level of broiler chickens supplemented with antibiotic growth promoter (AGP) or protected organic acids (OA) and essential oils (EO) [P(OA + EO)] raised under field conditions. Values are means of 40 replicates.

The analysis of the composition of the cecal microbiota revealed that the most abundant phylum observed was Firmicutes, followed by Bacteroidetes, Proteobacteria, and Verrucomicrobia, without differences between the treatment groups (*P* > 0.05). At a downstream taxonomic level (Figure 4A), it was observed that the microbiota was dominated by members of the family *Ruminococcaceae* (48.8%), *Lachnospiraceae* (20.9%), *Bacteroidaceae* (7.4%), and *Lactobacillaceae* (5.5%), without difference between the treatment groups (*P* < 0.05). The supplementation of P(OA + EO) increased the abundance of the family *Rikenellaceae* (3.02 vs. 3.61%; *P* = 0.05) and tended to increase *Porphyromonadaceae* (1.66 vs. 2.34%; *P* = 0.08) and *Anaeroplasmataceae* (*P* = 0.07).

At the genus level (Figure 4B), it was observed that the most abundant genera were *Faecalibacterium* (30.0%), *Ruminococcus* (11.0%), *Bacteroides* (11.0%), *Oscillospira* (10.5%), and *Lactobacillus* (8.3%). It was observed that P(OA + EO) supplementation increased the frequency of *Parabacteroides* (2.5 vs. 3.5%; *P* = 0.040), and *Coprobacillus* (1.1 vs. 1.4%; *P* = 0.040) and tended to increase the frequency of the genus *Dorea* (2.1 vs. 2.5%; *P* = 0.070) and decrease *Bacteroides* (12 vs. 10.1%; *P* = 0.100).

### Prevalence of Antimicrobial Resistance Genes

The prevalence of AMR genes is shown in Table 2. It was observed that the removal of AGP and inclusion of P(OA + EO) reduced (*P* < 0.05) the frequency of four AMR genes, related to gentamicin (three genes), and aminoglycoside (one gene), and tended to reduce (*P* = 0.09) the prevalence of one tetracycline resistance gene.

**TABLE 2:** Prevalence of antimicrobial resistance genes in the cecal microbiota of broiler chickens supplemented with antibiotic growth promoter (AGP) or protected organic acids (OA) and essential oils (EO) [P(OA + EO)] raised under field conditions.

**Treatment**	**aadA**	**aac3-VI**	**aac3-VI**	**tetB**	**aph(3)IA**	**pcoD**	**Sull**	**groEL**	**dfr17**
AGP	47.5	10	22.5	25	47.5	42.5	87.5	42.5	55
P(OA + EO)	22.5	5.0	2.5	10.0	25.0	42.5	62.5	47.5	57.5
SEM	0.08	0.08	0.08	0.08	0.06	0.08	0.04	0.07	0.06
*P*-value	0.02	0.008	0.008	0.09	0.04	0.92	0.74	0.58	0.72

*Values are the number of samples positive for the specific gene over the total number of samples (40).*

*aadA, aminoglycoside; aac3-VI, gentamicin; tetB, tetracycline; aph(3)IA, gentamicin; pcoD, copper; sull, sulfa; groEL, chaperone; drf17, trimethoprim; SEM, standard error of mean.*

## Discussion

The purpose of the present study was to evaluate the effect of P(OA + EO) to replace AGP in the diets of broiler chickens raised under field conditions. Overall, it was observed that the supplementation of P(OA + EO) improved intestinal health as demonstrated by the histopathological analyses and the additional biomarkers evaluated herein. Furthermore, the supplementation of P(OA + EO) prevented shifts in the microbiota diversity and composition due to the removal of AGP and had positive immunomodulatory effects on the host. Even though the growth performance data was not analyzed, we demonstrated, by using different methodologies, some of the mechanisms by which P(OA + EO) improves the intestinal health of broiler chickens.

It has been widely discussed in the last years that the non-antibiotic anti-inflammatory effects of AGP may explain, at least in part, the beneficial results on the growth performance of broilers (Niewold, 2007). Broom (2017) cited another mechanism of action of AGP that may be responsible for the improvement in growth performance of chickens. The author reported that AGP (sub-inhibitory concentrations) influences the dynamics of the intestinal microbiota which in turn reduces the release of pro-inflammatory molecules, reflecting a change in the immune system-microbiota interface (Broom, 2017). Therefore, one can assume that AGP and its alternatives, such as OA and EO, act directly on the host, or by modulating the intestinal microbiota and its metabolic functions, changing, for example, the production of short-chain fatty acids (SCFA), and antimicrobial peptides, that would exert influence on the immune system of the host. Metagenomics analysis of the intestinal microbiota would be essential to determine the changes of different signaling pathways.

The histopathological assessment used herein allows the study of inflammatory events that may damage the intestine (Kraieski et al., 2016; Belote et al., 2019). Sanches et al. (2020) showed that broiler chickens develop a microscopic basal enteritis, even in the absence of challenge, throughout their life, which is characterized by an increased inflammation of the epithelium and lamina propria, immature enterocytes proliferation, epithelium thickness, congestion and goblet cells proliferation. Additionally, Belote et al. (2019) demonstrated that the ISI histological analysis had a strong correlation with the growth performance, and that the higher the ISI score the worse the performance of broiler chickens. This methodology was also used in the present study as a tool to evaluate the degree of intestinal inflammation. The supplementation of P(OA + EO) reduced most of the parameters evaluated, and the total ISI score, indicating a lessened intestinal inflammatory response associated with its supplementation when compared to AGP supplemented chickens. Furthermore, the reduction of the serum concentration of CALP is another indicator of attenuated intestinal inflammation by P(OA + EO). Dal Pont et al. (2021) showed that broiler chickens with the highest CALP concentration in the blood at 14 days also had the highest total ISI score in the jejunum. At 28 days, the highest CALP concentration in the excreta was positively correlated to the highest ISI total score in the duodenum and ileum (Dal Pont et al., 2021).

Another unique methodology applied in the present study, which has very limited number of publications in chickens, is the chicken-specific cytokine array. With this analysis, we were able to measure the concentration of several cytokines and acute phase proteins (APP) in the serum of broiler chickens raised under field conditions. We demonstrated that the dietary inclusion of P(OA + EO) significantly reduced the serum concentration of IL-12, IL-16, and PTX3 and numerically reduced IFN-gamma. IL-12 is a pro-inflammatory cytokine that induces Th1-type immune response typically associated with the production of IFN-gamma (Degen et al., 2004), and, therefore, its elevated blood concentration may be linked to chronic inflammation. In such scenario, nutrients must be diverted from production parameters to support the immune response, while it can suppress feed intake and nutrient availability for growth and induce catabolism of host tissues (Broom and Kogut, 2018). IL-16 is described as a chemoattractant (Wigley and Kaiser, 2003) which also possess pro-inflammatory characteristics (Saleh and Al-Zghoul, 2019). In agreement to our study, Swaggerty et al. (2020) have demonstrated that heterophils isolated from chickens fed microencapsulated OA and EO showed increased degranulation and oxidative burst response compared to those isolated from chickens fed control diet, showing that they are able to modulate the immune system, and therefore, alter the susceptibility to disease.

Additionally, PTX3 is an indicator of early inflammation recently described in chickens and expressed by a variety of tissues (Burkhardt et al., 2019). It was demonstrated that PTX3 is stimulated by IFN-gamma and is up-regulated by a number of viral and bacterial diseases, such as infectious bursal disease, avian pathogenic *Escherichia coli* (APEC), and Marek’s disease (Burkhardt et al., 2019). The authors concluded that PTX3 is a potential biomarker to monitor the inflammatory status of poultry flocks. Even though IL-10 was not statistically different between both groups (*P* = 0.07), it is a regulatory cytokine that suppresses pro-inflammatory cytokines (He et al., 2011). In fact, it has been shown that IL-10 inhibits IFN-gamma synthesis by mitogen-activated lymphocytes (Rothwell et al., 2004). Therefore, the reduction of serum IL-10 concentration, may be due to a lessened inflammatory status, as observed by the reduction of IL-12, IL-16, and PTX3, which clearly shows that the supplementation of P(OA + EO) is beneficial in attenuating chronic inflammation faced by modern strains of broiler chickens.

We observed very slight changes on the makeup of the cecal microbiota following the removal of AGP, which shows that the supplementation of P(OA + EO) maintained the balance of the microbiota similar to AGP fed chickens. One cannot discard all the factors that influence the intestinal microbiota of chickens, including feed, environmental conditions, sex, age, among others (Kers et al., 2018), especially when field samples are analyzed. In terms of bacterial families, *Rikenellaceae* was the only one that was significantly increased when the birds were supplemented with P(OA + EO). *Rikenellaceae* and *Ruminococcaceae* increased in mice fed a high-fat diet, suggesting that the increase in the abundance of these bacteria occurs in obese animals (Kim et al., 2012). Moreover, in humans, this bacterial family has been found to be increased in healthy compared to non-alcoholic fatty liver disease patients (Jiang et al., 2015). On the other hand, *Parabacteroides* and *Coprobacillus* were increased by the P(OA + EO) supplementation. To better understand the core microbiota and its association with the growth performance of antibiotic-free commercial broiler chickens Johnson et al. (2018), using a large dataset, reported that *Parabacteroides* and *Coprobacillus* in the cecum were positively correlated with body weight of 21- to 28-days old broilers. The samples of the present study were collected from 25- to 28-days old birds, which falls within the former range, and shows beneficial effects of the P(OA + EO) supplementation for antibiotic-free chickens. According to a bacterial meta-analysis of the cecal microbiota of chickens, *Coprobacillus*, even though in a small abundance, has been found in most of the analyzed studies (Chica Cardenas et al., 2021).

It has been well-documented in the literature that the indiscriminate use of antimicrobials may accelerate the emergence of AMR genes in bacteria from poultry production, which may also lead to economic losses, derived from the expenditure on ineffective antimicrobial (Nhung et al., 2017). In the present study, we demonstrated that replacing AGP by P(OA + EO) reduced the prevalence of AMR genes in the cecal microbiota, while keeping a balanced microbiota composition and diversity. The possibility of reducing AMR genes by the use of P(OA + EO) must be further studied, but it can be seen as a potential tool to re-establish the antimicrobial sensitivity to improve the action of antibiotics when poultry flocks need to be treated against a disease.

## Conclusion

Overall, the inclusion of P(OA + EO), and removal of AGP, in the diets of commercially raised broiler chickens beneficially changed the phenotype of the jejunum, as shown by the lowered ISI scores which characterizes an improved intestinal health. Additionally, P(OA + EO) significantly reduced the serum concentration of several inflammatory biomarkers, while keeping the diversity and composition of the cecal microbiota similar to AGP fed chickens. It can be concluded that P(OA + EO) improves the health of the intestinal mucosa by directly acting on the host and maintaining the balance of the microbiota and reducing the frequency of AMR genes, showing its potential to be widely used in field conditions. Further studies should look at the effects of these molecules on the metagenome of the intestinal microbiota.

## Data Availability Statement

The data presented in this study were deposited and made publicly available in the NCBI SRA under accession PRJNA743867 (https://www.ncbi.nlm.nih.gov/sra/PRJNA743867; release date: 2022-08-01).

## Ethics Statement

Ethical review and approval was not required for the animal study because the samples of the present study were collected from a field study at a commercial farm. Therefore, an ethical review was not necessary. Written informed consent was obtained from the owners for the participation of their animals in this study.

## Author Contributions

CB: conceptualization, sample collection, laboratory analyses, and writing and editing. LL, ES, and MK: conceptualization, data analysis and interpretation, and reviewing and editing. JO and DD: reviewing and editing. CE: samples collection, and reviewing and editing. NB: laboratory analyses, and reviewing and editing. All authors contributed to the article and approved the submitted version.

## Conflict of Interest

CB, LL, JO, DD, and ES are employed by Jefo Nutrition, Inc. The remaining authors declare that the research was conducted in the absence of any commercial or financial relationships that could be construed as a potential conflict of interest.

## Publisher’s Note

All claims expressed in this article are solely those of the authors and do not necessarily represent those of their affiliated organizations, or those of the publisher, the editors and the reviewers. Any product that may be evaluated in this article, or claim that may be made by its manufacturer, is not guaranteed or endorsed by the publisher.

## Footnotes

1 Swaggerty, C. L., Bortoluzzi, C., Eyng, C., Lee, A., Dal Pont, G. C., and Kogut, M. H. (2021). Development of alternatives to feed antibiotics: interactions at the gut level and their impact on host immunity. *Adv. Exp. Med. Biol.*

2
https://www.ncbi.nlm.nih.gov/sra/PRJNA743867
